# Supplementary material for: Development of an Ex Vivo Functional Assay for Prediction of Irradiation Related Toxicity in Healthy Oral Mucosa Tissue
Source: Int J Mol Sci. 2024 Jun 28;25(13):7157. doi: 10.3390/ijms25137157 (PMC11241643; doi:10.3390/ijms25137157)
Supplement: Supplementary file 1 [file ijms-25-07157-s001.zip › ijms-3032843-supplementary.pdf]

Supplementary Material

Supplementary Material and Methods

2.3 Tissue irradiation – Proton setup

For the proton setup, tissue slices were put in empty 70 µm Nylon cell-strainers (Falcon, ref. 352350) and fixed with three drops of ice-cold hydrogel (Mebiol, gel concentration 10% [w/v], ref. PMW20-1001), which was previously dissolved in culture medium without growth factors. After hydrogel solidification for 3 minutes at 37°C, cell strainers with slices inside were moved to 6-well-plates (Sarstedt, ref. 83.3920.005), which were previously filled with 12.5 ml medium of 37°C. These 6-well-plates were closed with 3D printed Formlabs clear resin lids (Formlabs 3 printer) and immediately put in the incubator at 37°C. Irradiation happened in the next 60 minutes after the setup was finished.
20 minutes after irradiation (based on radiation safety measurements), cell strainers were moved to cold PBS for two minutes which was necessary to get tissue slices out of the hydrogel. Thereafter, cell strainers were put into 6-well plates with warm 4.5 ml cell culture medium and in the incubator on a Stuart SSM1 mini orbital shaker at 60 rpm.

2.6 Immunohistochemistry (IHC) staining

Antibody	Dilution	Species	Company	Clone
IL1-beta	1/3200	Rabbit	Abcam	Polyclonal
CD45	3,24 ug/ml	Mouse	Ventana	UCHL-1
CD27	1/500	rabbit	Atlas	polyclonal

Table S1: Immunohistochemistry antibody information.

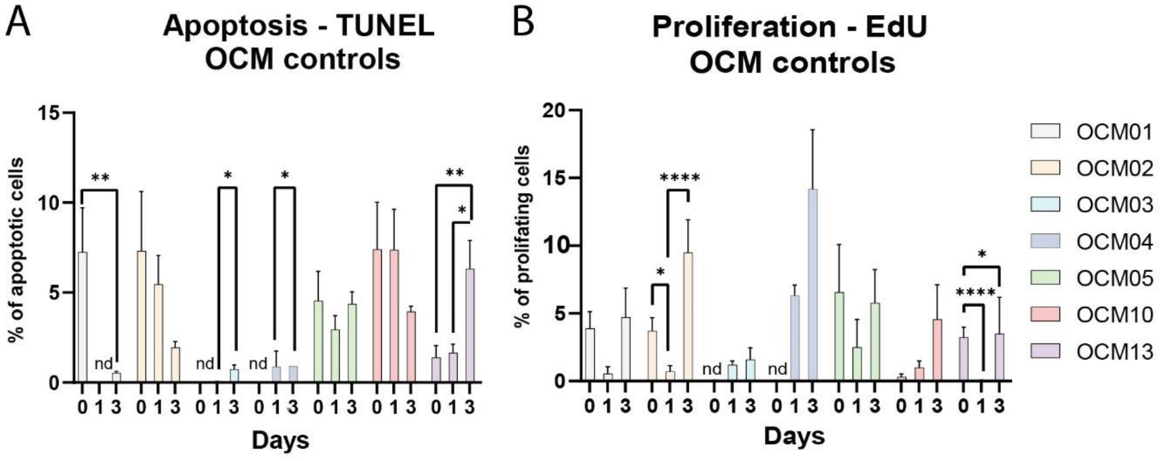

Figure S1: Healthy mucosa tissue slices maintain viability for several days in culture. (A) Apoptosis values of control conditions per sample at day 0 (2 hours), day 1 (24 hours) and day 3 of ex vivo culture. (B) Proliferation values of control conditions per sample at day 0, day 1 and day 3 of ex vivo culture. All graphs represent mean of ≥3 FoV per sample and error bars depict SEM. All samples were analyzed with Kruskal Wallis test or Mann Whitney test (if condition is missing) per sample. * $p < 0.05$, ** $p < 0.01$, *** $p < 0.001$, **** $p < 0.0001$.

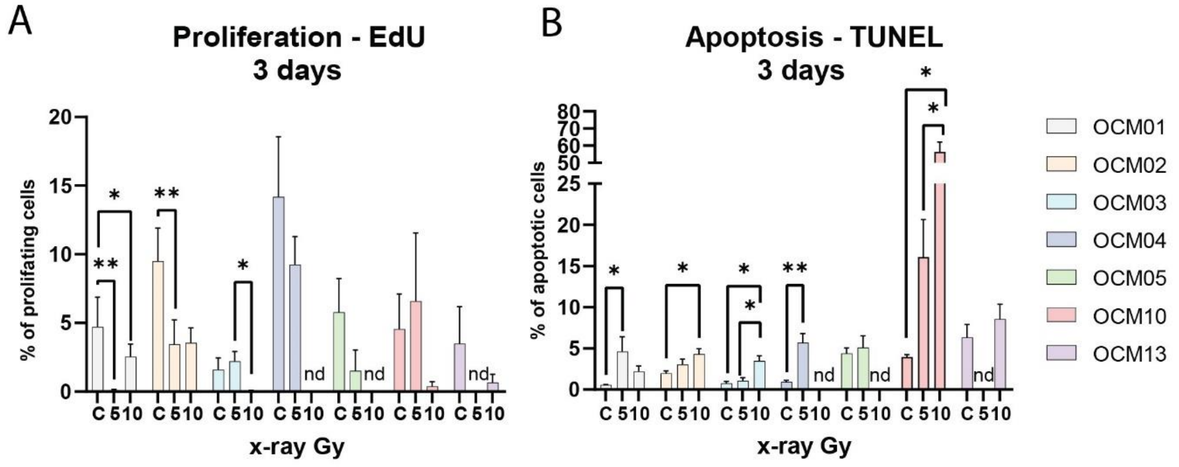

Figure S2: Healthy mucosa displays dose-dependent response to X-ray irradiation. (A) Proliferating cells per sample and treatment condition upon three days of ex vivo culture after 5 Gy and 10 Gy X-ray irradiation. (B) Apoptotic cells per sample and treatment condition upon three days of ex vivo culture after 5 Gy and 10 Gy X-ray irradiation. All graphs represent mean of ≥ 3 FoV per sample, and error bars depict SEM. All samples were analyzed with Kruskal Wallis test or Mann Whitney test (if condition is missing) per sample. * $p < 0.05$, ** $p < 0.01$.

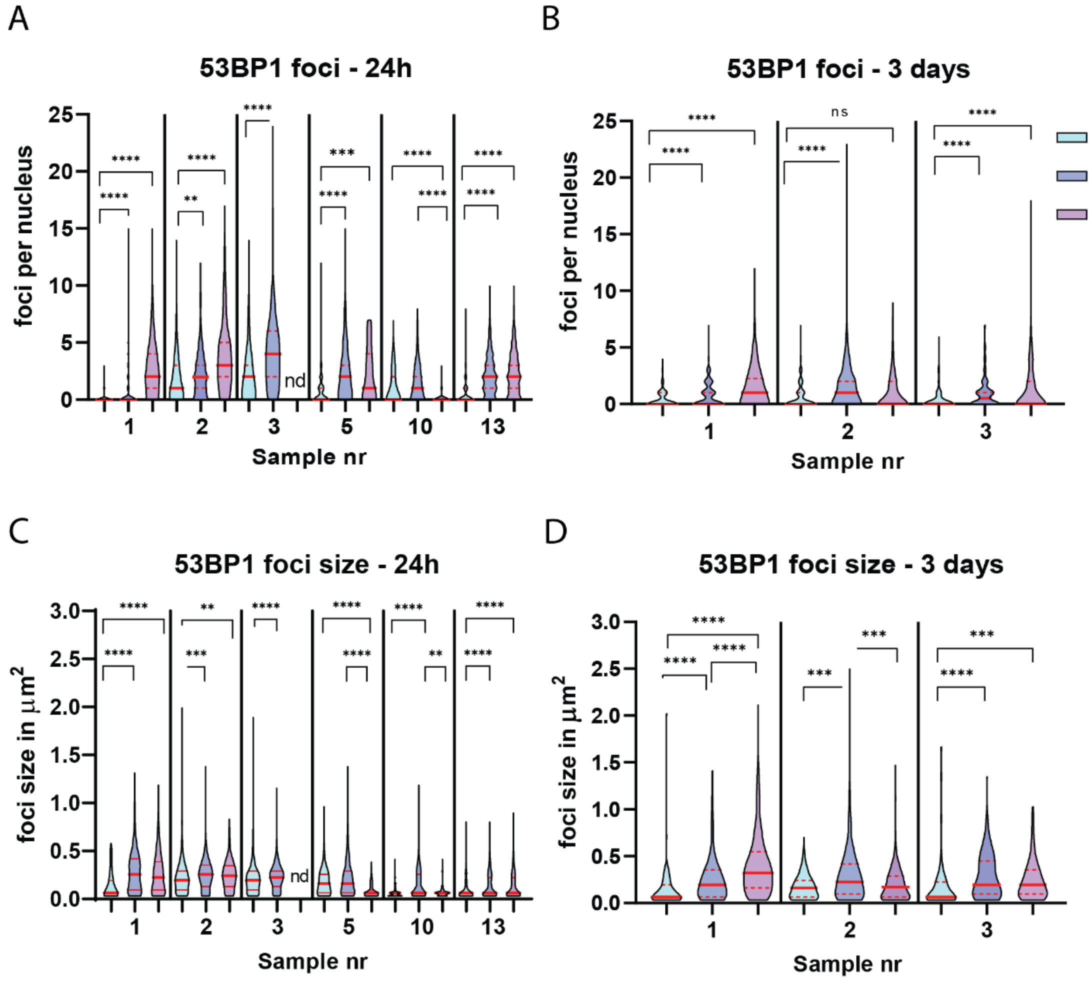

Figure S3: Residual DNA damage foci after irradiation of normal mucosa. Number of 53BP1 foci per nucleus per sample and treatment condition upon 24 hours (A; $n = 6$) and three days (B; $n = 3$) of ex vivo culture after 5 Gy and 10 Gy X-ray irradiation. 53BP1 foci size per sample and treatment condition upon 24 hours (C; $n = 6$) and three days (D; $n = 3$) of ex vivo culture after 5 Gy and 10 Gy X-ray irradiation. All graphs represent median of all nuclei and foci analyzed (≥100 nuclei per sample for foci number, ≥75 foci per sample for foci size) and 1st and 3rd quantile (dashed lines). Kruskal-Wallis and Dunn's multiple comparison test was used for significance. ** $p < 0.01$, *** $p < 0.001$, **** $p < 0.0001$.

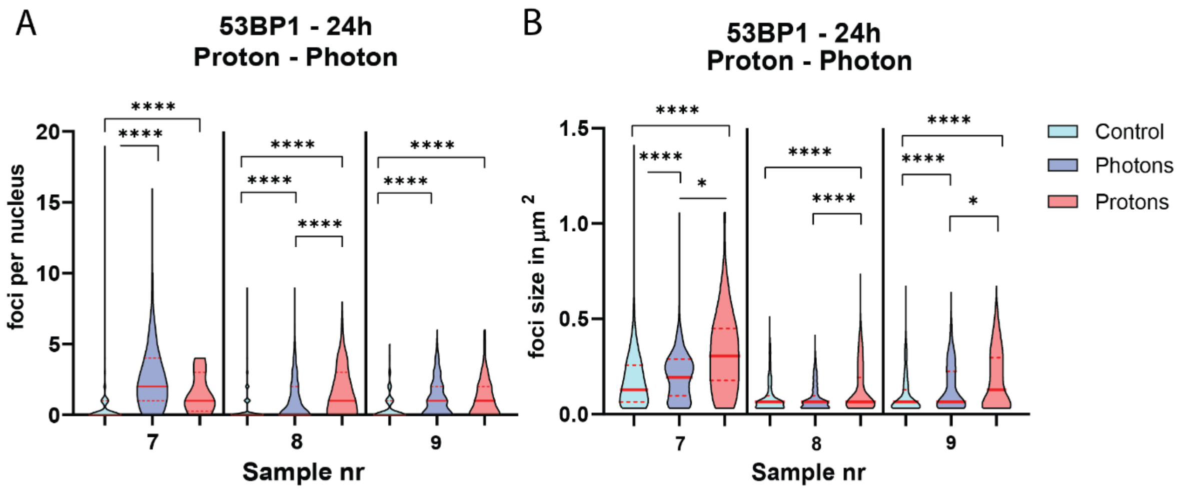

Figure S4: Proton irradiation of mucosa tissue slices. 53BP1 foci per nucleus (A) and foci size (B) per sample in control, 5 Gy X-ray and 5 Gy proton irradiated conditions after 24 hours of ex vivo culture (n = 3). Graphs represent median of all nuclei analysed (≥ 50 nuclei per sample, ≥75 foci per sample for foci size) and 1st and 3rd quantile (box) as well as minimum and maximum values (error bars). Kruskal-Wallis and Dunn's multiple comparison test was used for significance. * $p < 0.05$, **** $p < 0.0001$.

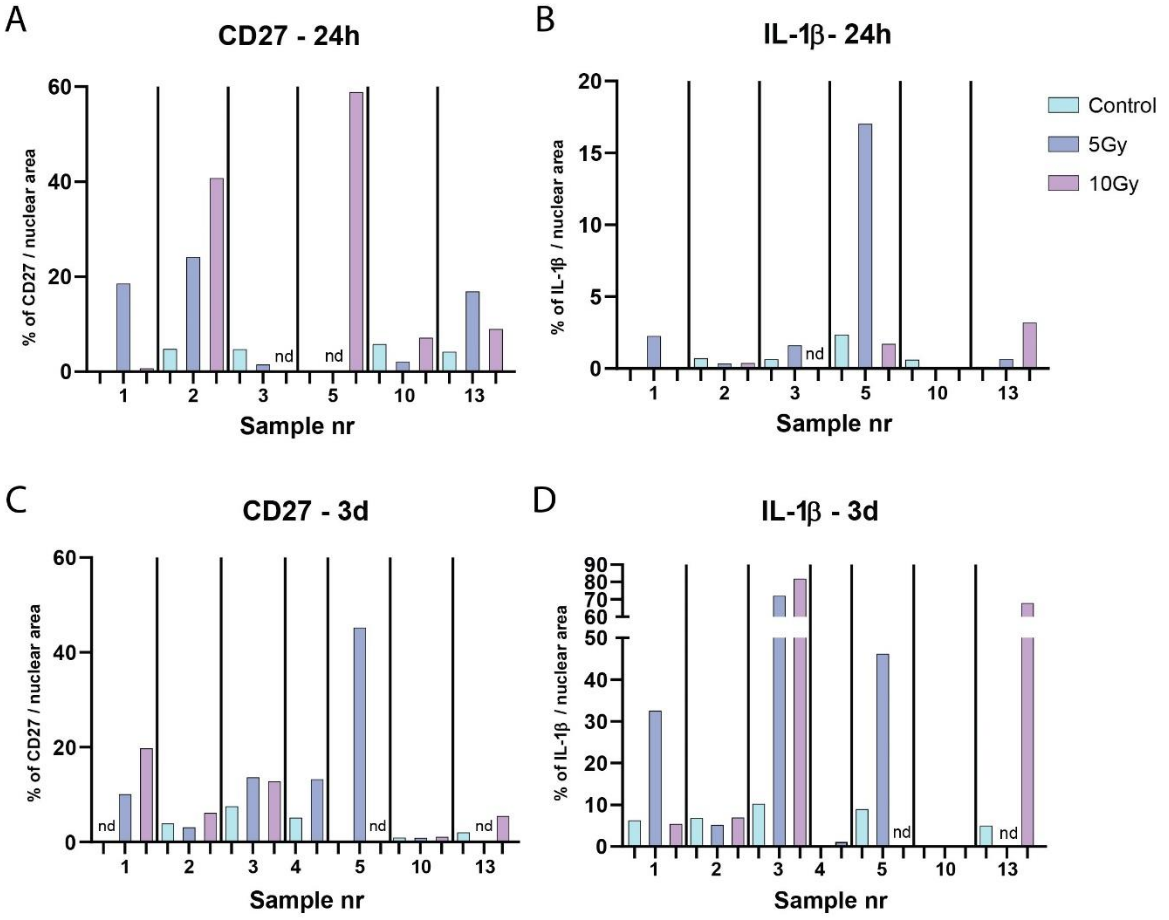

Figure S5: Immune cells express inflammation inducing factors upon irradiation ex vivo. Area measurement of CD27 (A) and IL-1β (B) signal per nuclear area upon 24 hours of ex vivo culture (n = 6) after 5 Gy and 10 Gy X-ray irradiation. Area measurement of CD27 (C) and IL-1β (D) signal per nuclear area upon three days of ex vivo culture (n = 7) after 5 Gy and 10 Gy X-ray irradiation. All graphs display mean, representing 1 - 3 FoV.
